# Feasibility of Using Strength Measures, Including Peak Inspiratory Flow, for Routine Monitoring in Case Management Patients Aged 65 and over

**DOI:** 10.3390/geriatrics5030059

**Published:** 2020-09-21

**Authors:** Nicola Barnes, Bronagh Walsh, Dinesh Samuel

**Affiliations:** Faculty of Environmental and Life Sciences, University of Southampton, Highfield Campus, Southampton SO17 1BJ, UK; Nicola.barnes@port.ac.uk (N.B.); B.M.Walsh@soton.ac.uk (B.W.)

**Keywords:** grip strength, peak inspiratory flow, peak expiratory flow, reliability, feasibility, acceptability

## Abstract

Peak inspiratory flow (PIF) is a portable, relatively new method for measuring respiratory function and indirect muscle strength; the feasibility of its routine clinical measurement is unknown. To investigate the acceptability, reliability and short-term stability of PIF, alongside the established measures of peak expiratory flow (PEF) and grip strength in community dwelling case management patients. Patients were tested in a sitting position, initially on two occasions, one week apart; seven patients having repeated measures taken on a further four occasions over a seven-week period. The best of three attempts for all measures were recorded. Reliability was tested using intra-class correlation coefficient (ICC), standard error of measurement (SEM), minimal detectable change (MDC) and Bland–Altman analysis. Eight patients aged 69–91 years (mean age 81.5 ± 7.7 years; 5 males) participated. For between-day reliability using the first two time points, one week apart the ICCs (3,1) were 0.97, 0.98 and 0.99 for PIF, PEF and grip strength respectively; using all five time points resulted in ICCs of 0.92, 0.99 and 0.99 respectively. Bland–Altman plots also illustrated a good level of agreement across days. Feedback on the acceptability of the measures was gathered from patients. PIF, PEF and grip strength showed excellent reliability and acceptability. Whilst excellent reliability was observed over the seven-week period, the occurrence of clinically significant symptoms and adverse events in the presence of unchanging PIF, PEF and grip strength, suggests that the measures may not be suitable to identify patients with multiple health conditions entering a period of acute decline.

## 1. Introduction

Muscle strength has repeatedly been shown to be a good predictor of future health and function in the long term, with a loss of muscle strength associated with declines in both physical and mental health including chronic disease, cognition, depression and frailty, as well as being part of normal ageing [1,2,3]. Measures of physical activity and function have been identified as potentially useful clinical markers of health [2,4,5]. However, simple, portable measures of strength have yet to be embraced as part of routine general clinical practice in the community setting, and the value of monitoring muscle strength in the shorter term as a predictor of health is unknown. Measures such as strength may be particularly useful in patients experiencing multiple disease states, where it is the cumulative effect of these, as in deficits in frailty, which is important. More generic areas of monitoring such as muscle strength are of additional benefit where typical disease specific symptoms may not be present, present too subtly or present too late to allow timely intervention.

Case management patients are such a patient group, experiencing a combination of the physiological changes of normal ageing and specific LTCs, along with pathological changes associated with LTCs in general, such as chronic inflammation, which often result in atypical symptoms which complicate diagnosis and treatment, making usual diagnostic methods and tools such as symptom report less useful [6]. Enabling the identification of such functional declines in the presence of atypical presentation and complex symptoms may aid timely and proactive interventions, avoiding adverse outcomes and unplanned service use. Whilst measuring functional ability is often subjective and reliant on patient self-report, muscle weakness, which may result in a lack of function, and vice versa, has been repeatedly identified as an objective measure that lends itself to assessment in the clinical setting of interest. Lack of guidance and evidence regarding what should be monitored and assessed to enable timely interventions are likely to be contributing to the disparity between case management services across the country and inequality in its provision [7]. Muscle strength may be especially useful as its measurement can be objective, simple and non-invasive [2]. Existing evidence focuses on longer-term changes (years) in muscle strength, health and function; there is a well reported reduction in muscle strength expected with normal ageing from 65 years onwards of 1–2% per annum, but changes over a shorter time period (week and months) are unknown [1,8]. There is also insufficient information on which measure to use. The most commonly investigated measures of strength are of limb strength; the correlation between grip strength and lower body and trunk strength has been demonstrated in various populations, including in healthy middle to old aged populations, with the former lending itself to use in the community [9,10]. Grip strength has been strongly linked to morbidity, mortality, functional performance, disability, cognition and lower quality of life [2,3,10,11,12,13]. Grip strength has been shown to be reliable and has been suggested as the single best measure of age-related change in muscle strength [14,15]. The possible role of grip strength for predicting subsequent health and risk of hospitalization in community dwelling older people has been repeatedly highlighted, accompanied by recommendations for further research into the value of measuring change in the shorter term [16,17]. Grip strength lends itself to clinical use in primary care, being portable, reliable and easy to perform [18]. Its practicality and ability to provide an indication of physical performance in younger old (i.e., 65–74 years of age) community dwelling individuals has been observed [19]. Practically, the equipment, although portable, is still relatively heavy and bulky to be carried around by a clinician and has a significant cost associated with it, both in the initial purchasing and routine calibration. 

Respiratory muscle strength in frail, older people has been less well studied than grip strength [20,21]. However, there are inexpensive, simple, reliable measures of respiratory function such as peak expiratory flow (PEF), that are a common part of clinical practice in the management of patients with respiratory disorders (they are rarely used in those without) and these could easily be included in regular monitoring of patients if they were found to be a good proxy indicator of strength and associated risk of declining health and function. Respiratory tests are often limited by their lack of specificity to measure either pulmonary function or respiratory muscle strength alone. Despite this lower pulmonary function and respiratory muscle strength have been associated with ageing, mobility disability and total mortality in the elderly, although some contradictory findings have been reported regarding their predictive value for all-cause mortality [22,23,24]. Respiratory muscle function is important for whole body functional performance and it may be useful to assess the changes in this specific function as one age. The portable respiratory measure, peak inspiratory flow (PIF), has recently become commercially available, whilst peak expiratory flow is already utilized in clinical practice for measuring respiratory function. Few published normative data for PIF exist; PIF is rarely reported in those without chronic pulmonary disease leading to a lack of published reliability studies in older individuals without respiratory impairment [25]. The likely presence of a chronic respiratory disease, e.g., chronic obstructive pulmonary disease (COPD), present in approximately half of the patients receiving case management in England [7], and the lack of specificity of measures of respiratory muscle strength, may reduce the usefulness of respiratory measures of strength. However, the interplay between pulmonary function and respiratory muscle strength may mean that a reduction in a respiratory measure is still a valid indicator of reduced muscle strength [23]. The addition of the measurement of PIF may help produce a more robust picture of respiratory muscle strength than PEF alone.

The present study aimed to investigate the reliability and short-term stability and patient acceptability of PIF, alongside the more established PEF and grip strength measures in older participants, in order to inform a preliminary assessment of their potential clinical usefulness.

## 2. Methods

Eight community dwelling case management patients (69–91 years, 5 males) were studied, data collection occurring in patients’ homes. Patients were recruited via community case management services from two Primary Care Trusts. Sample size calculation suggested that a sample size of 12 was sufficient to identify a 25% change in the muscle strength measures, 10 a change of 35% and 8 a 45% change. The lower sample size was utilised due to challenging recruitment over a 12-month period, during which three different recruitment methods were utilised. All methods involved patients being initially identified via a collaborative approach between the case management team and researcher, then followed by an invitation pack provided (a) by hand by their case management team, or (b) by post from the researcher following an initial phone call by the case management team, or (c) posted to the patient direct by their case manager.

Inclusion Criteria for participation were men and women aged 65 years of age and over receiving case management.

Exclusion Criteria for participation were: enrolled in any other research study; a condition that precluded consent, i.e., a significant cognitive impairment or lack of capacity to consent, unable to communicate without significant aids; a condition that precluded participation/ collection of data, i.e., significant emotional distress or psychotic illness active within the last six months, receiving end of life care, current illness that would preclude data collection, including acute exacerbation of COPD or asthma, upper limb pathology that would limit participation, e.g., recent bone fracture; high risk patients with regards to lone working safety.

Respiratory function was measured using PIF (Clement Clarke In-Check Oral, Clement Clarke International, Harlow, UK) and PEF (Clement Clarke Mini-Wright Standard Range, Clement Clarke International, Harlow, UK), grip strength measured by JAMAR^®^ Hand Dynamometer (Performance Health International Ltd., Sutton-in-Ashfield, UK),. Participants were tested in a sitting position, by a single operator, on a minimum of two and maximum of five occasions, over a maximum seven-week period, with one to two weeks between assessments. Following an initial trial attempt, the best of three maximal efforts for PIF, PEF and grip strength were recorded.

For grip strength measurement participants were tested in an upright sitting position, with their shoulder adducted and neutrally rotated, elbow flexed at 90°, forearm in neutral position, and wrist between 0° and 30° dorsiflexion and between 0° and 15° ulnar deviation. The JAMAR^®^ Hand Dynamometer handle position was standardized at the second handle position from the inside for all participants, and participants’ dominant hand used.

PEF and PIF maneuvers were performed in a sitting position, patients asked to sit as upright as possible. PIF participants were asked to fully exhale then inhale forcefully through their mouth, taking a short sharp breath in of around one second in duration. PEF participants were asked to take a deep breath in then exhale/blow as hard and fast as they could through the mouthpiece.

Feedback was provided to allow technique to be improved if needed and deviation recorded. Although it is standard practice when obtaining PEF and PIF measurements to have the patient stand, recent evidence does not support the need to stand, demonstrating no significant difference in PEF values whether sitting or standing [26,27].

Physical activity and function data were gathered via researcher administered Physical Activity Scale for the Elderly (PASE), Barthel Index (BI) and the Vulnerable Elders Scale (VES-13). PASE is a 12-item scale measuring physical activity over the previous seven days; scores were calculated as per standard scoring instructions, using weights and frequency values for each type of activity providing a score between 0 and 400 or more [28]. BI and VES-13 were both included as measures of functional ability. BI covers 10 aspects of function and is widely used in community services to aid assessment of a person’s ability to live independently, but due to its relative insensitivity to small changes in functional ability that may be expected in the patient group it was used alongside VES-13 [29,30]. VES-13 was developed as a function based screening tool for community dwelling older adults to identify people at risk of health deterioration [31], and includes functional aspects seen in frailty scales [32] and domestic aspects not included in the BI. The VES-13 is targeted towards the vulnerable old, relevant to the patient group.

Changes in patients’ health and medical history including symptoms, unplanned health service use and medication were obtained by patient report each visit.

Strength measure reliability was tested by intra-class correlation coefficient (ICC; 3,1), standard error of measurement (SEM), minimal detectable change (MDC) and Bland and Altman analysis. Data were analyzed using SPSS v.21/22 (IBM, New York, NY, USA).

Likert scales were utilized to gain feedback from the participants on the acceptability of the muscle strength measures.

The study was approved by the Southampton B National Health Service Ethics Committee (12/SC/0313) on 27 July 2012. Informed consent was obtained from all individual participants included in the study. All procedures performed in studies involving human participants were in accordance with the ethical standards of the institutional and/or national research committee and with the 1964 Helsinki declaration and its later amendments or comparable ethical standards.

## 3. Results

### 3.1. Participant Characteristics

The baseline characteristics of the sample are shown in Table 1. Males and females were of similar age, with both male’s and female’s body mass index (BMI) within the overweight category. Diagnosed LTCs and the taking of regular prescribed medication were common amongst the study participants. Adverse health outcomes were reported by participants during the study period; two reported falls and one reported an unplanned hospital attendance and admission.

PIF, PEF and grip strength values fell just below or at the lower end of expected normal ranges for male and female participants, correspondingly the group mean level of physical activity measured by the PASE fell below published norms [28,33,34,35]. Despite small numbers, positive correlations between the three muscle strength measures were suggested; a non-significant relationship between grip strength and PEF (Pearson’s two tailed correlation *r* = 0.66, *p* = 0.071), and a significant correlation between grip strength and PIF (*r* = 0.893, *p* = 0.03). A non-significant correlation between grip strength and PASE was also suggested (*r* = 0.266, *p* = 0.524).

The sample demonstrated a high variability in levels of independence, with Barthel Index scores ranging from very low to very high (range 20–100; maximum achievable score, i.e., highest functional ability is 100) and VES-13 scores (range 2–10; maximum achievable score, i.e., highest functioning is 0, while a score over 3 identifies a person as at higher risk (four times that of a person scoring 3 or less) of functional decline and death in the following one to two years [31]). Six of the eight participants had a score above 3 on the VES-13. Levels of physical activity, as measured by PASE, varied widely (range 7–146; maximal achievable score, i.e., most active is 400). A non- significant positive correlation was suggested between BI and grip strength (*r* = 0.391, *p* = 0.338), with a corresponding non-significant negative correlation between VES-13 and grip strength (*r* = −0.671, *p* = 0.069).

### 3.2. Reliability of Respiratory Measures and Grip Strength

Intra-rater reliability of tests performed on the first two data collection days, one week apart, estimated using ICCs, showed all measures as having an ICC indicative of an acceptable clinical measure (Table 2).

**Figure 1:**
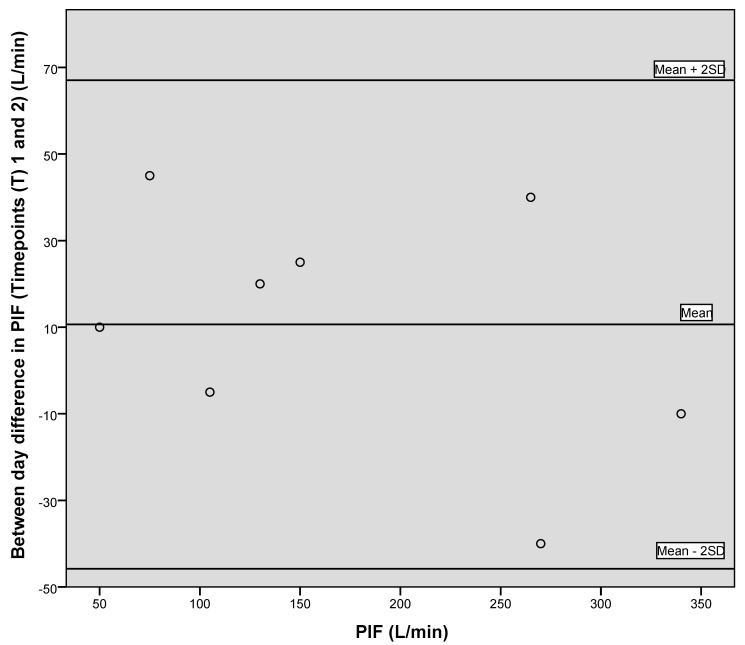
Bland–Altman plot showing between day variation for peak inspiratory flow (PIF).

**Figure 2:**
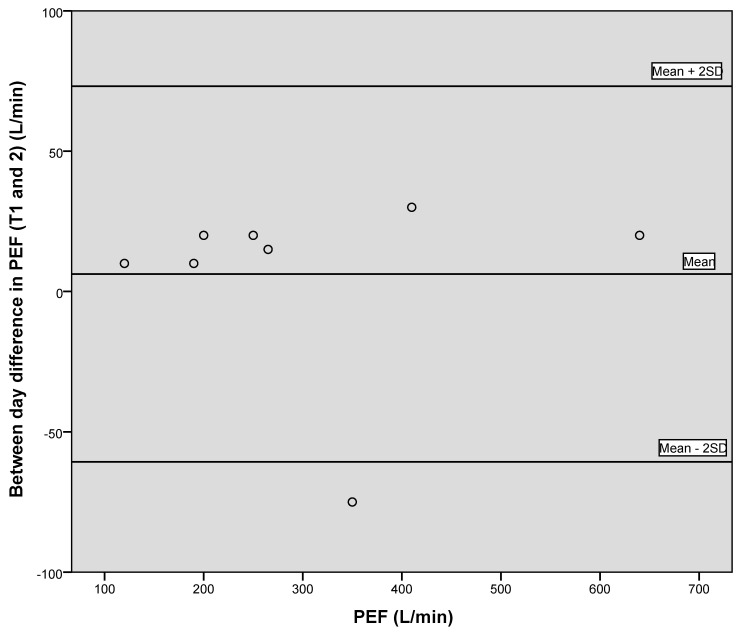
Bland–Altman plot showing between day variation for PEF.

**Figure 3:**
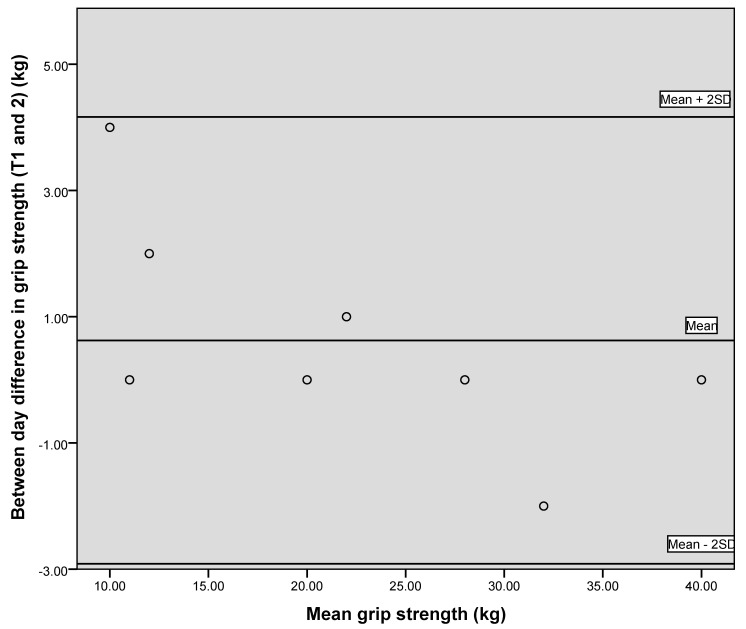
Bland–Altman plot showing between day variation for grip strength.

Figure 1, Figure 2 and Figure 3 illustrate the between-day variation observed for the first two data collection points, one week apart, for PIF, PEF and grip strength, and demonstrate a generally good level of agreement.

Table 3 indicates that all three measures demonstrated stability over the seven-week data collection period.

**Figure 4:**
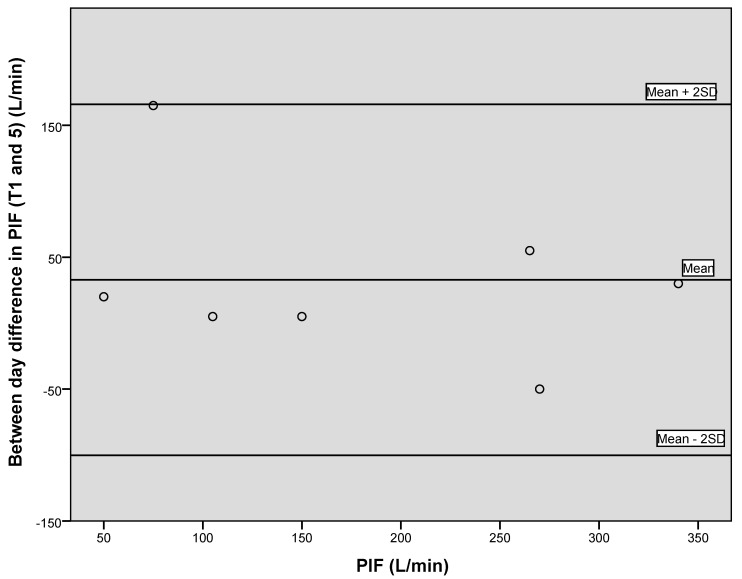
Bland–Altman plot showing between day variation for PIF between time point 1 and 5.

**Figure 5:**
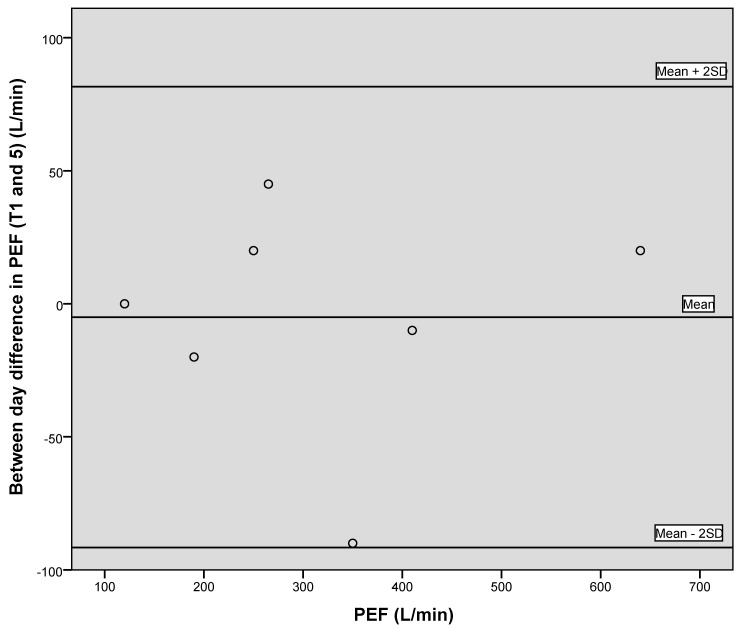
Bland–Altman plot showing between day variation for PEF between time point 1 and 5.

**Figure 6:**
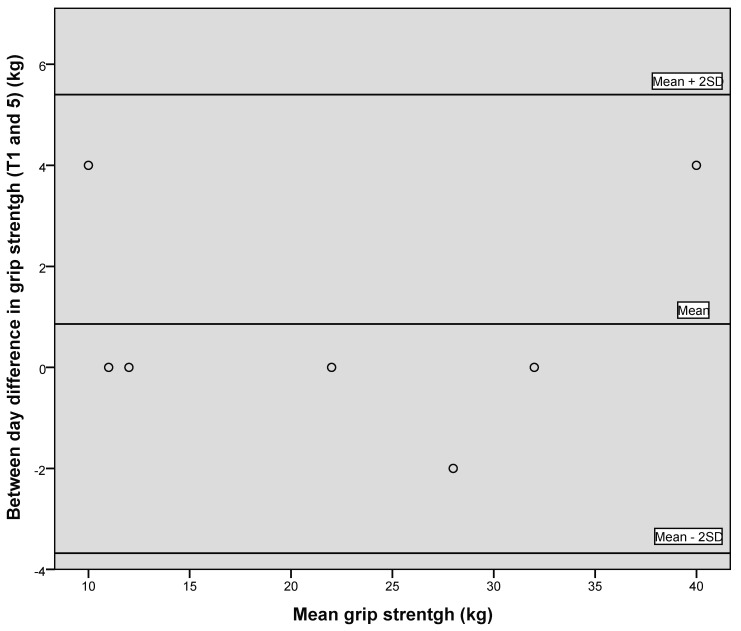
Bland–Altman plot showing between day variation for grip strength between time point 1 and 5.

Figure 4, Figure 5 and Figure 6 illustrate via Bland–Altman plots the between day variation observed for grip strength, PEF and PIF, between the first time point and final time point, seven weeks apart. Due to the small sample size, conclusions should be treated with caution. However, the plots demonstrate that good agreement between days was generally observed.

Both Bland–Altman plots for PIF (Figure 1 and Figure 4) illustrate that in the majority of participants, an increase in readings from time point one was observed, suggestive of a learning effect.

### 3.3. Acceptability of Measures of Strength

Patients generally found grip strength to be the most acceptable strength measure (Table 4). Whilst grip strength was repeatedly identified as the easiest to understand how to perform and to perform, it was rated the least comfortable to perform. Complete strength readings were recorded on all occasions, with no unexpected side effects or problems observed.

## 4. Discussion

Reliability estimates for PIF, PEF, and grip strength in the present study suggested that all could be measured consistently, accurately and acceptably in case management patients aged 69 years and over by a single operator, and, furthermore, remained stable over the seven-week testing period. The ICCs for all measures were above the recommended 0.90 to ensure reliability for clinical measurements [36], and Bland–Altman plots demonstrated good agreement between days. The reliability estimates for all strength measures were in line with previously published work [37,38,39]. Slightly lower reliability was observed with PIF, which may be indicative of a learning effect. Despite the small numbers, correlations were suggested between all muscle strength measures and the physical activity and functional measures in the expected directions, supporting the agreement between all.

If considering the measures as clinical tools in patients without measure specific conditions/symptoms, e.g., COPD, asthma, rheumatoid arthritis, the high level of stability observed over the seven-week data collection period suggests that repeated measurement of grip strength, PEF and PIF would not be beneficial over the short to medium term, as the measures appear to remain stable over this period, despite patient reports during the study period of adverse events including falls, unplanned hospital attendance and admission. However, further investigation with a larger sample size would be required to confirm this and to explore the relationship between muscle strength and clinically relevant outcomes, but the data strongly suggests that this may not be a fruitful avenue for future research due to the overall stability of these measures. It may be more beneficial to focus future research on the predictive nature of a one off measure of muscle strength. The data did demonstrate the feasibility of all of the measures of strength, PEF, PIF and grip strength in such a patient group.

### Study Limitations

Recruitment of case management patients was challenging, whilst ample numbers of patients on case lists were evident, there was a difficulty in accessing these patients and gaining a response. A low response rate to the recruitment strategies was observed (e.g., 30% response rate to the postal recruitment drive) along with a higher than expected refusal rate (e.g., 75% for combined phone and postal recruitment drive), and higher than expected rate of loss at screening (up to 50%). Lack of response to the initial planned recruitment strategy led to amendments to the protocol, which included alternative recruitment methods, a reduction in the frequency of follow up visits, and a reduction in the length of the data collection period. Recruitment of older people into research has been shown to be difficult, with refusal rates of up to 54%, exclusion rates of up to 49%, and drop-out rates of 5–37% [40]. It was also apparent that a substantial proportion of patients on the case lists utilized met the study exclusion criteria, by either receiving end of life care or having significant cognitive impairment. This study demonstrated further the difficulties faced by researchers in recruiting older patients into research studies. Researchers wishing to recruit similar community-dwelling patient groups should consider the study design, including all aspects of recruitment and data collection protocols and locations.

The study involved participants performing the respiratory measures in a sitting position, in variation to the manufacturers recommended protocol. This testing position was used due to concerns regarding the ability of patients to be able to safely stand and perform the measures. Recent evidence demonstrating no significant difference in PEF values whether sitting or standing, helped inform the change to testing position [26,27]. It should however be considered that sitting may have impacted on both PEF and PIF, resulting in below maximal values being obtained. All patients were asked to sit up right whilst performing PEF and PIF; in practice, there was variability in the ability of patients to do this, which is likely to have restricted inspiratory and expiratory movements, resulting in lower values. Whilst this was expected to have minimal impacted on the ability to answer the research questions on reliability, as the same procedure was used for all measurements allowing comparison, it may have lowered the group norms.

## 5. Conclusions

In conclusion, the results from this feasibility study indicate that PIF, PEF and grip are reliable and acceptable measures in older adults aged 65 years and over; however, their stability over the seven-week data collection period despite adverse health events occurring suggests that routine monitoring over the short term may prove fruitless.

## Figures and Tables

**Table 1:** Summary baseline characteristics of participants.

Characteristics	Males (*n* = 5) Mean ± Standard Deviation (SD)	Females (*n* = 3) Mean ± SD
Age (years)	82.8 ± 8.8	79.3 ± 6.4
BMI (kg/m^2^)	27.3 ± 1.8	29.2 ± 4
No. of reported regular prescribed medication	4.4 ± 2.9	5.7 ± 3.9
No. of reported diagnosed LTCs	2.2 ± 1.2	2.3 ± 1.9
Grip strength (kg)	29.2 ± 11.5	16.7 ± 3.1
PEF (L/min)	410.0 ± 163.6	188.3 ± 46.5
PIF (L/min)	283.0 ± 76.9	116.7 ± 35.1
BI	89.5 ± 18.5	64.2 ± 34.3
VES-13	5.8 ± 3.6	6.2 ± 1.2
PASE	81.3 ± 57.7	33.5 ± 4.4

**Table 2:** Intra-rater reliability of grip strength, peak expiratory flow (PEF) and PIF.

Measures	Mean ± SD	ICC (95%Confidence Interval (CI))	SEM	MDC
Grip strength (kg)	22.4 ± 10.6	0.991 (0.954–0.998)	1.01	2.80
PEF (L/min)	306.3 ± 165.6	0.980 (0.902–0.996)	23.42	64.92
PIF (L/min)	180.3 ± 102.8	0.967 (0.847–0.993)	18.67	51.75

**Table 3:** Between day variation (all 5 time points) in grip strength, PEF and PIF.

Measures	Mean ± SD	ICC (95%CI)	SEM	MDD
Grip strength (kg)	23.2 ± 10.7	0.988 (0.963–0.998)	1.17	3.24
PEF (L/min)	313.0 ± 164.4	0.988 (0.964–0.998)	18.01	49.92
PIF (L/min)	194.7 ± 100.0	0.923 (0.794–0.984)	27.75	76.92

**Table 4:** Acceptability of measures of muscle strength to patients.

Questions 1–4 Likert Scale 1–5, Where 1 Is Strongly Disagree, to 5 Strongly Agree (Mean Score)	PEF	PIF	Grip Strength
1. “It was easy to understand what I had to do”	4.0	3.9	5.0
2. “It was easy to do”	3.9	3.6	4.5
3. “It was comfortable to do”	4.9	5.0	4.4
4. “I would recommend the test to anyone”	5.0	5.0	5.0
5. Tests’ ranking in order of preference (1 to 3, where 1 is most preferred to 3 is least preferred):			
Mean	1.8	1.9	1.8
Median	2	2	1
Mode	2	2	1
Time taken, range in minutes, to complete 3 repetitions for PEF, PIF and grip strength	0–6	0–4	0–5

## Abbreviations

LTCs	Long term conditions
PEF	peak expiratory flow
PIF	peak inspiratory flow
COPD	chronic obstructive pulmonary disease
PASE	Physical Activity Scale for the Elderly
BI	Barthel Index
VES	Vulnerable Elders Scale
ICC	intra-class correlation coefficient
SEM	standard error of measurement
MDC	minimal detectable change
T	time point
